# Mr. Noble, on the Yellow Fever

**Published:** 1806-01

**Authors:** A. Noble

**Affiliations:** H. M. S. Amelia, Tortola


### To the Editors of the Medical and Physical Journal.

GENTLEMEN,

CONCEIVING that the following account of a successful practice employed in the treatment of Yellow Fever, lately occurring on board of His Majesty's Ship Amelia, may be of service in directing the practice of others; and being convinced that the bold, yet rational measures here described, will in similar cases save a number of lives, which, when left to nature, or treated by placebos, would certainly be lost, I hope it will meet your approbation to insert it in your Journal.

His Majesty's Ship Amelia went into English Harbour, Antigua, in the beginning of July to refit: she had a number of repairs to undergo, which exposed the men to great heat and fatigue; and it was now the most unhealthy season of the year, when the sun being perpendicular, rendered the heat excessive, and the breezes neither refreshing nor frequent; when heavy rains, and dead, hot calms were continually alternating: these causes in a fortnight having produced a sufficient predisposition, the inflammatory, ardent, or yellow fever, began to make its appearance. In some, the fever commenced with symptoms of debility, followed within twelve hours by increased action in every part of the system. In others, no precedent debility showed itself, but the commencement was sudden, and violent. Violent head-ach, particularly referred to the forehead, intense heat of skin, giving a burning sensation to the fingers, strong, hard pulse, an affection of the stomach, painful on pressure, and inducing constant nausea or vomiting, pain in the limbs and loins, and a peculiar inflamed appearance of the eye, easier remarked than described, were the characteristic symptoms of the disease.

All these symptoms were more or less violent in different cases; and this did not depend upon constitution, or habit of body, so much as might have been expected; sometimes a spare, delicate man would have symptoms of the greatest re-action; and the following case will show that the contrary also was experienced.

John Lewis was a strong, robust, hard working man; his station was captain of the hold, where frequent great exertion is required, and intense heat often borne. His head-ach was slight when attacked, and continued so; his

 pulse pulse was quick and soft, his stomach much affected, and a far greater prostration of strength was present, than is commonly met with in this fever, but no heat of skin ever appeared, it was dry and rather below the natural temperature; on the second day, a low, muttering delirium came on, and he died on the third. It seems probable that in this case the powers of life, or excitability of the system, was destroyed by the causes of fever, without first, as usual, producing a violent general increased action.

Nine months before the period of which I write, the Amelia had suffered much from the same fever; upwards of eighty men died, including the captain, surgeon, and most of the other officers; and I remarked, that although a number of those who before had the fever were again taken ill, the disease appeared in a milder form. After the ship left English Harbour, and the men became less exposed to fatigue, and breathed a cooler and purer air, though the predisposing causes still continued powerful enough to assist in producing the disease, it soon put on a milder appearance. Some are of opinion that the fever is contagious, others think differently; my observation leads me to believe, that the fear and depression of spirit occasioned by a number being taken ill, powerfully assisting in the production of the disease, has led to the former opinion.

The remedy to which I have principally trusted, and to which I owe the salvation of numbers, is cold bathing. The patient was put into a tub of salt water, and soused over head repeatedly, until it would have been dangerous to have kept him longer in the water, until his skin became perfectly cold, and he was ready to faint. By one such immersion in very violent cases, nothing was gained, but a great deal in milder ones; in the course of half, or a whole hour in the former, the heat, oppression at the præcordia, and general uneasiness, were as great as ever; the immersion was then immediately repeated, and continued every hour or oftener if the heat of skin was greatly above the natural standard; I regret that I had no thermometer to mark down the particular degrees the heat arrived at. The way I conceive this remedy to act, is not by giving a sudden shock to the system, but simply by abstracting heat. From particular circumstances I was prevented from renewing the bath to several of the sick in the night time, as I could have wished; to these I had a wet sheet applied around the body with the best effects, and either taken off, and dipped in cold water as it got warm, or cold cold water poured over it. Acting upon this principle I never hesitated about bathing a patient, while the skin was moist, or covered with perspiration; for though perspiration cools the surface, cold bathing does it sooner, and more effectually, and every moment is of consequence in preventing the great heat from producing incurable debility, or rather from entirely destroying the vital powers. In violent cases of fever, the bath was sometimes required sixteen or twenty times; in milder cases, the heat would at first take some hours before it returned, that period got gradually longer, and after four or five bathings the fever would be entirely subdued.

But even to the cold bath, powerful as it is, I could not entirely trust, in very violent cases of yellow fever; it was necessary to employ another agent, which, conjointly, almost insured safety. I had been accustomed last war to trust every thing to the cold bath; I often experienced its excellent effects, but occasionally found it to fail, even when used in the powerful manner above described. The first opportunity I had of observing the good effects of mercury, in conjunction with the cold bath, was some months back at St. Kitts, under the humane and intelligent practice of Dr. Armstrong, whose chief study is to meliorate the sufferings of human nature, and whose chief pride is in his successful practice; I there saw the happiest result from the powerful combination of calomel and cold bathing; many cases apparently desperate were recovered, where the first forty-eight hours had been lost; and I felt a conviction that had I seen the practice sooner, I might have saved the lives of more than one. The sickness taking place in the Amelia soon after, gave me a large field for experiencing its good or bad effects, and the following is the result. Twenty very violent cases of fever kept on board the ship, and treated with calomel and cold bathing, all recovered. Thirty others, where the symptoms were milder, recovered by cold bathing only; out of twenty sent to Antigua hospital, some of which were bathed for the first twelve hours of the attack, where the heat of skin allowed of that practice, five died; and fifteen were returned in a state of convalescence, on our leaving the harbour.

I know that the use of calomel has been cried down by some, merely from the inert method in which they administered it: they give three or four grains every three or four hours; and long before, in this manner, the fever can be overcome, the powers of life are destroyed, and the

 fatal fatal debility supervenes; but when by large and frequent doses, the system is certainly affected in the first twenty-four hours, I never failed to observe the malignant and threatening symptoms lessened. And the best criterion I know of, to judge of the danger of the case, is by the facility, or difficulty, of affecting the system; where a fatal termination is greatly to be dreaded, and every part is in a torpid state, calomel will neither purge when combined with rhubarb, nor affect the mouth when given by itself. This remedy I conceive induces a different action in the system, or that the fever brought on by calomel destroys the yellow fever, upon the principle that no two different actions of malignant tendency can exist at the same time in the system. In those violent cases which I judged required the use of this remedy, to assist the cold bath, I gave ten grains of calomel every two hours; sometimes it was continued in this manner for the first twelve hours only, and sometimes for the first thirty-six; after that period I never found it necessary; but during this time it was always assisted by one, two, three, or four ounces of mercurial ointment.* I have seen Dr. Armstrong give seven drachms of calomel, and rub in three or four pounds of mercurial ointment in the course of three days; however, I never found occasion to use more than above related.

The infusion of Columba I think materially assists blisters to the stomach in removing the cause of vomiting, but blisters are indispensible, and when the vomiting still continued after one, a second was applied next day. As soon as the heat of skin was removed, I immediately employed the cold infusion of bark in small, frequent doses, and in the intervals Port wine, cold or hot, as best relished by the patient. It often happened that the vomiting still continued very troublesome, and then, though the first, second, and third doses immediately given after each other, were rejected, the fourth would be retained, from the exhaustion of the stomach's excitability; nor did I ever observe any harm from persisting in this manner, but, on the contrary, the stomach would gradually recover its tone, and the vomiting gradually disappear.

I am, &c.

A. NOBLE.

*H. M. S. Amelia, Tortola, Aug. 27, 1805.*

---

* These doses appear enormous to European practitioners. Ed.

